# Semi-Supervised Prediction of SH2-Peptide Interactions from Imbalanced High-Throughput Data

**DOI:** 10.1371/journal.pone.0062732

**Published:** 2013-05-17

**Authors:** Kousik Kundu, Fabrizio Costa, Michael Huber, Michael Reth, Rolf Backofen

**Affiliations:** 1 Bioinformatics Group, Department of Computer Science, University of Freiburg, Freiburg, Germany; 2 Centre for Biological Signalling Studies (BIOSS), University of Freiburg, Freiburg, Germany; 3 Institute of Biochemistry and Molecular Immunology, University Clinic, RWTH Aachen University, Aachen, Germany; 4 Department of Molecular Immunology, Max Planck Institute of Immunology, Freiburg, Germany; 5 Centre for Biological Systems Analysis (ZBSA), University of Freiburg, Freiburg, Germany; 6 Center for non-coding RNA in Technology and Health, University of Copenhagen, Frederiksberg, Denmark; University of Alberta, Canada

## Abstract

Src homology 2 (SH2) domains are the largest family of the peptide-recognition modules (PRMs) that bind to phosphotyrosine containing peptides. Knowledge about binding partners of SH2-domains is key for a deeper understanding of different cellular processes. Given the high binding specificity of SH2, in-silico ligand peptide prediction is of great interest. Currently however, only a few approaches have been published for the prediction of SH2-peptide interactions. Their main shortcomings range from limited coverage, to restrictive modeling assumptions (they are mainly based on position specific scoring matrices and do not take into consideration complex amino acids inter-dependencies) and high computational complexity. We propose a simple yet effective machine learning approach for a large set of known human SH2 domains. We used comprehensive data from micro-array and peptide-array experiments on 51 human SH2 domains. In order to deal with the high data imbalance problem and the high signal-to-noise ration, we casted the problem in a semi-supervised setting. We report competitive predictive performance w.r.t. state-of-the-art. Specifically we obtain 0.83 AUC ROC and 0.93 AUC PR in comparison to 0.71 AUC ROC and 0.87 AUC PR previously achieved by the position specific scoring matrices (PSSMs) based SMALI approach. Our work provides three main contributions. First, we showed that better models can be obtained when the information on the non-interacting peptides (negative examples) is also used. Second, we improve performance when considering high order correlations between the ligand positions employing regularization techniques to effectively avoid overfitting issues. Third, we developed an approach to tackle the data imbalance problem using a semi-supervised strategy. Finally, we performed a genome-wide prediction of human SH2-peptide binding, uncovering several findings of biological relevance. We make our models and genome-wide predictions, for all the 51 SH2-domains, freely available to the scientific community under the following URLs: http://www.bioinf.uni-freiburg.de/Software/SH2PepInt/SH2PepInt.tar.gz and http://www.bioinf.uni-freiburg.de/Software/SH2PepInt/Genome-wide-predictions.tar.gz, respectively.

## Introduction

Protein-protein interaction is a major area of biological science to understand transduction of cellular signals. One important function of protein-protein interactions is to mediate post translational modifications by binding of a protein domain with a short linear peptide [1]. Receptor tyrosine kinases (RTKs) are the largest kinase family that phosphorylate specific tyrosine residues in a protein and play a vital role in signal transduction by regulating a variety of essential cellular processes such as proliferation, differentiation, growth, migration, apoptosis and malignant transformation in metazoans [2]–[5]. There are two types of protein domains that recognize the phosphotyrosine (pTyr) residue in a linear peptide, namely src homology 2 (SH2) and protein tyrosine binding (PTB) domains [6], [7]. SH2 domains are structurally conserved protein domains containing a central 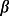 sheet flanked by 2 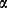 helices, normally found in intracellular signal transducing proteins [8], [9]. Previous study indicated that there are around 120 SH2 domains in 110 unique human proteins and each SH2 domain binds with distinct phosphopeptides [10]. There are some evidences that mutations in some SH2 domains can cause several human diseases like XLP syndrome [11], Noonan syndrome [12], X-linked 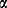-gammaglobulinemia [13] and basal cell carcinoma [14]. Researches using peptide libraries have shown that each SH2 domain binds with a specific subset of phosphopeptides [15]–[18]. Computational identification of SH2-domain specific binding to arbitrary phosphopeptides within a complex cellular system is an open challenge with high relevance.

Due to the high number of SH2-domains, one has to resort to high-throughput data for defining the binding specificity. Over the years several experimental approaches and associated computational prediction methods have been developed to identify in-vitro binding specificity of human SH2 domains.

One of the most popular tools is *Scansite*, which was developed by *Yaffe et. al.* in 2003 [19] and is based on position specific scoring matrices (PSSMs) derived from chemically synthesized peptide array libraries [19], [20]. More recently, a similar approach called *SMALI* has been published by *Li et al.* in 2008 [21], which is also based on PSSMs derived from a slightly different library approach called OPAL (oriented peptide array libraries) [22], [23]. In another recent work (*DomPep*), the authors propose a linear SVM based method to predict domain-peptide interactions [24].

PSSM models, as used by Scansite and SMALI and SVM models, as used by DomPep are essentially linear models that are not capable of reflecting the complex dependencies between amino acid positions. Furthermore, PSSM based tools induce models based only on confirmed interactions (positive interactions) but don’t exploit the information from negative interactions. In order to incorporate more complex interactions and thus to improve prediction accuracy, other approaches used structural information of SH2-peptide complexes and energy models derived thereof. Examples are comparative molecular field analysis (CoMFA) [25], FoldX algorithm [26], [27] and others [28]–[30]. Unfortunately these approaches are computationally very expensive and depend on solved structures, which are given only for few SH2-peptide complexes. One exception is *Wunderlich et al.*, who presented an energy model that can be used for almost all human SH2 domains [31]. However the good performance reported seems to be due to some over-training issues (see Results Section).

Previous research showed that the correlations between different ligand positions take important role in the binding specificity of the SH2 domains [32]. In recent years, polynomial kernels have been successfully applied to the prediction of DNA-protein interactions [33]. In this paper, we propose domain specific non-linear models for SH2-peptide interactions that are based on support vector machines. As the complexity of the model increases so does the required number of training instances. While modern high-throughput techniques seem to be the perfect solution to the data requirements, they have other issues. The first problem is that techniques like pool oriented peptide arrays (such as [22], [23]) do not test individual peptides but pools of peptides with common properties. In a second phase, individual peptides are tested with separate methods. Thus, while these approaches provide information about real interactions (positive data), they cannot reliably be used to assess the lack of a domain-peptide interaction. A similar situation occurs with many high-density peptides arrays where affinities are not reported. Other high throughput approaches like microarrays do report affinities (e.g. [34] and [35]) and thus can be used to assess the lack of strong interaction. These approaches suffer, however, from a low signal to noise ratio and therefore produce results that are often inconsistent. For example, in one microarray experiment [34] found that the number of interactions between 11 peptide sequences extracted from protein ErbB1 and 85 SH2 domains is 37, while in similar settings in another microarray experiment [35] found three times as many interactions.

This state of affairs leads to a great imbalance between the available information on positive vs. negative interaction data. Such an imbalance constitutes a severe problem when fitting a predictive model. For example, for some SH2 domains, the information on real interactions can be up to 15 times more abundant than the information on the lack of interaction. It is known that in these conditions predictive systems produce suboptimal results.

We propose a semi-supervised iterative approach to tackle all these issues. We devise a non-linear support vector machine (SVM) model for each of 51 human SH2 domain. These models can successfully exploit the information on the dependency between position specific amino acids. To tackle the problem of data imbalance, we developed a simple yet effective approach to make the best use of various types of experimental interaction measurements. To be more specific, we first extract an initial high quality dataset from high density peptide arrays and micro array experimental results. In a second step, the data is rebalanced using a self-training strategy.

We show that our approach performs significantly better than state-of-the art SH2-peptide interaction prediction tools. Furthermore, when applying it on high quality hand-curated SH2-peptide interaction data from PhosphoELM database [36], we achieved higher True Positive Rate (TPR) in comparison to PSSM models (SMALI) and energy model. In addition we perform a genome-wide analysis and find interesting insights of biological relevance. Finally, we make our models and genome-wide predictions freely available to the scientific community.

## Results and Discussion

### Model

Our approach takes in input peptide sequences that have been previously aligned, and, as it is common in literature, it is based on amino-acid positional features. The alignment phase induces a global position system where the phosphotyrosine residue is given position 0. Differently from most approaches though, we propose to model complex non-linear dependencies between the amino-acid positional features.

Previous studies showed that residues in the close vicinity of the phosphotyrosine are highly predictive for SH2 domain-peptide binding [19], [21], [31]. For example it is known that the SH2 domain of CRK binds peptides where amino acid Leu or Pro is in position +3, however the presence of other amino acids (i.e. His, Arg, Ala, Pro) in position +1 and +2 can inhibit the interaction. [32]. Thus, we followed the literature and restricted the peptide sequence to 6 specific positions, namely we extracted all the amino acids ranging from 2 upstream to 4 downstream of the phosphotyrosine residue. A peptide is therefore mapped into a binary vector 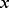 living in a 20×6 = 120 dimensional space (as the central amino acid is always a phosphotyrosine, it is not informative and it is not included in the encoding), that is, for each position, we reserved 20 dimensions (one for each amino acid) and encoded the amino acid type with a 1 in the corresponding dimension and 0 elsewhere.

For the predictive model, many popular approaches, such as SMALI [21], are based on PSSMs. We note that these methods are essentially linear models and cannot therefore model arbitrary functional dependencies between amino acid positions.

Here we propose three ways to improve over PSSM models: 1) upgrading the system from linear to non-linear, 2) making the system more robust using *regularization* techniques, and 3) making an effective use of both interaction information (positive examples) and non-interaction information (negative examples) by dealing with the imbalanced issues.

More in details, non linear models allow to express decision rules that can take into consideration complex functional dependencies between amino acid positions. For example it could be important to differentiate between the situation where we have the co-occurrence of two or more amino acids and the situation where one has independent occurrences of the same amino-acids in different peptides. For example consider a case where the presence of amino acid Asn in position +2 alone is not sufficient to guarantee the interaction and neither is the presence of amino acid Lys in position −1. However if these amino acids are occurring in their respective positions at the same time then the binding occurs. Note that there can be different instances of this situation, such as two or more amino acids can have an non-additive effect as described in the example, or two or more amino acids can exclude each other etc. In order to model this non-linear dependencies (but at the same time control the complexity of the model), we upgrade to polynomial kernels(for details, see *Methods*, Subsection *Regularized Non-linear Support Vector Machine*). Note that the degree of the polynomial kernel is optimized via cross-validation and hence, a simpler *linear* model can still be chosen for some SH2 domains when it offers better performance.

The second improvement is to employ regularization techniques to avoid overfitting. Albeit there are many different ways of dealing with this problem, we adopt the strategy that has been championed in support vector machines. The basic idea of regularization is to minimize the complexity of the model by adding a penalty to discount the cumulative size of the parameters. To be more precise, the complexity of the model depends on the degree of the polynomial kernel (since this determines the number of parameters) and on the cumulative size of the parameter vector in the SVM (for details, see again *Methods*, Subsection *Regularized Non-linear Support Vector Machine*).

**Figure 1:**
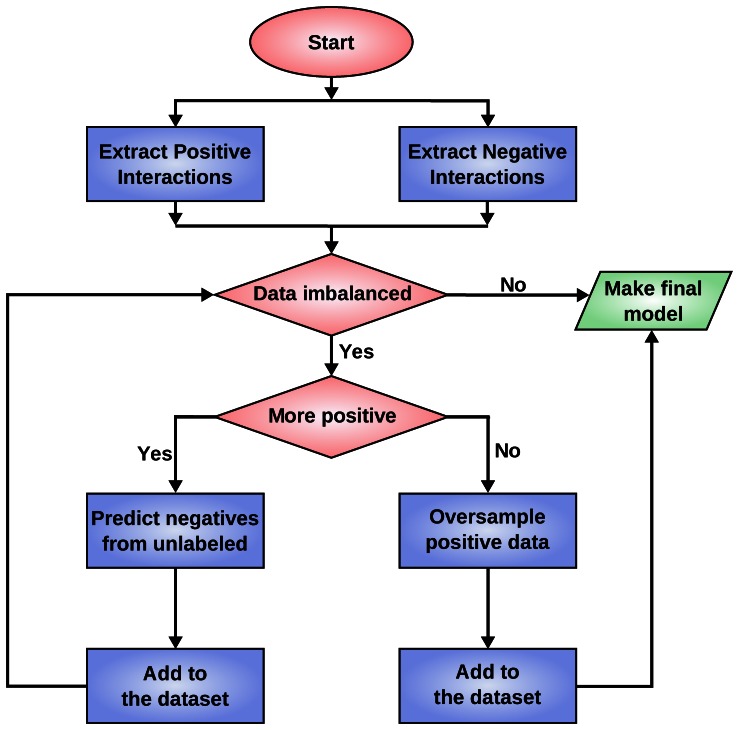
Flowchart for the iterative negative data filtering. An initial high quality dataset is extracted from experimental evidence. If the negatives are in excess (right branch) then we simply duplicate the positive instances. If the positives are in excess (left branch) then we make an initial model using over-sampled negatives; this model is then used to score all the available peptides. Those that are more confidently predicted as negatives are added to the dataset. The procedure is iterated until a balanced dataset is reached. The final model is computed on the balanced dataset.

Using the polynomial kernel, we achieve a higher SH2-peptide interaction modeling flexibility. As a consequence of this increased flexibility, we need a larger number of training instances. Notwithstanding the availability of dataset derived by high-throughput techniques, we still suffer from lack of reliable negative data (see *Materials and Methods* section). This is the main cause for the high imbalance: for some SH2 domains, information on real interactions can be up to 15 times more abundant than information on the lack of interactions (see Table S1). It is known that in these conditions predictive systems produce suboptimal results (for further details see subsection *Modeling and learning issues: a short review on the imbalanced dataset problem* in the *Materials and Methods* Section). To mitigate these issues, we propose the pipeline depicted in Figure 1. The main idea is to bootstrap from a smaller set of reliable negative instances and only select peptides that we are highly confident to yield negative interactions. Specifically, the pipeline works as follows: 1) an initial high quality, experimentally verified, dataset is extracted from high density peptide arrays and micro array results; 2) data is rebalanced using a self-training strategy with polynomial SVM; 3) model selection is performed to select the best model complexity for each specific SH2 domain. The key points here are the a) rebalancing strategy, and the b) self-training phase. For rebalancing we use over-sampling in order not to throw away valuable information as would be done with under-sampling strategies. The self-training is a straightforward yet effective wrapper technique that can be applied to any classifier. It consists in an iterative procedure where at each stage the current model predicts the class label over the unsupervised material. In the next training phase the class labels for the most confident predictions are used. The procedure can then be iterated. In our case the confidence is scored as the distance from the discriminative hyper plane.

### Evaluation

In order to assess the expected predictive performance of our approach, we have performed two types of experiments: (i) a cross-validation and random splitting on combined data from three sources: a peptide array library data (dataset I) and two microarray datasets (dataset II and dataset III); moreover (ii) we performed a validation experiment using a manually curated SH2-peptide interaction dataset (dataset IV) (see *Methods* for details).

We compare the performance against two state-of-the-art approaches: 1) a tool based on PSSMs and 2) an energy model based on interaction maps. The first tool, called SMALI [21] is available for 76 SH2 domains and is based on the same peptide representation we use (i.e. −2 to +4 amino acids with pTyr in 0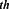 position). The second tool [31] is an energy model based on different types of interaction maps where only the positions of amino acids found to be in contact are used.

#### Predictive Performance Evaluation Setup

On each SH2 domain we evaluate the predictive performance of our approach with a stratified 5 fold cross-validation. Here the data set is split into 5 equal parts, which are all used in turn as test sets. The remaining 4/5 of the data is used in turn as training material. The hyper-parameters, i.e. the polynomial degree, the trade-off between fitting and smoothing cost parameter 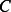, are determined on a ten-fold cross-validation. The whole cross-validation procedure is then repeated 5 times. Using a repeated random split with 75% of the data for training and the remaining 25% for testing, we obtain performance values which are comparable to those obtained in the cross-validation setting (see Figure S1).

**Figure 2:**
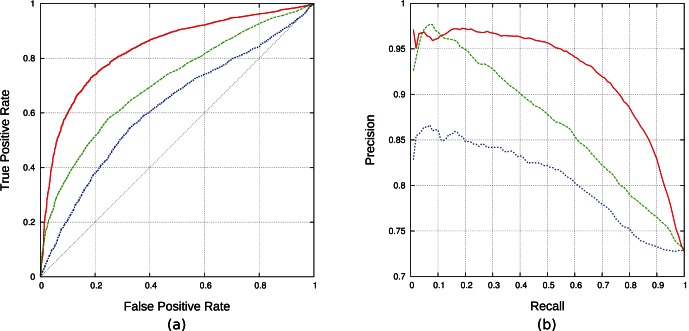
Comparison of AUC ROC and precision-recall curve of three different approaches. (a) Showing the comparison of the AUC ROC for the SVM performance (solid red line), the SMALI performance (dashed green line) and the performance of energy model (dotted blue line). This figure clearly indicates the SVM performance with 0.83 AUC ROC is significantly higher than the SMALI and energy model approaches with 0.71 and 0.62 AUC ROC respectively. (b) Showing the comparison of the precision-recall curve for the SVM performance (solid red line), the SMALI performance (dashed green line) and the performance of energy model (dotted blue line). In this case the SVM performance with 0.93 precision-recall curve is higher than the SMALI and energy model approaches with 0.87 and 0.81 precision-recall curve respectively.

We compute the area under the ROC curve (AUC ROC) and the area under the precision and recall curve (AUC PR) (see Figure 2). Additionally, we report sensitivity, specificity with standard deviation per domain for different treatments of negative data in Table 1, where the first column refers to no imbalance treatment, the second refers to a random re-balancing strategy and the last refers to the proposed iterative self-training strategy.

**Table 1:** Comparison of specificity and sensitivity.

Domains	Original		Random re-sample		Neg Semisup	
	Specificity	Sensitivity	Specificity	Sensitivity	Specificity	Sensitivity
ABL1	0.54 ±0.08	0.84 ±0.1	0.84 ±0.17	0.45 ±0.09	0.75 ±0.14	0.68 ±0.14
ABL2	0.53 ±0.33	0.88 ±0.09	0.81 ±0.32	0.35 ±0.1	1 ±0	0.55 ±0.17
APS	0.64±0.11	0.82±0.08	0.88 ±0.13	0.55 ±0.16	0.67 ±0.14	0.74 ±0.13
BCAR3	0.44 ±0.29	0.72 ±0.07	0.7 ±0.1	0.38 ±0.15	0.55 ±0.18	0.56 ±0.09
BLK	0.55 ±0.14	0.92 ±0.04	0.8 ±0.11	0.63 ±0.07	0.7 ±0.19	0.78 ±0.11
BMX^†^	0.74 ±0.05	0.79 ±0.09	–	–	–	–
BRDG1^†^	0.76 ±0.11	0.82 ±0.08	–	–	–	–
BTK	0.54 ±0.11	0.78 ±0.08	0.86 ±0.1	0.36 ±0.16	0.88 ±0.1	0.64 ±0.2
CRK	0.67 ±0.16	0.97 ±0.03	0.96 ±0.1	0.68 ±0.12	0.85 ±0.13	0.89 ±0.05
CRKL	0.63 ±0.17	0.92 ±0.05	0.96 ±0.09	0.71 ±0.13	0.94 ±0.09	0.8 ±0.12
CTEN	0.89 ±0.08	0.7 ±0.08	–	–	–	–
E105251	0.57 ±0.16	0.83 ±0.07	0.92 ±0.08	0.43 ±0.06	0.69 ±0.09	0.75 ±0.06
E109111	0.65 ±0.29	0.89 ±0.04	0.88 ±0.07	0.55 ±0.11	0.81 ±0.13	0.67 ±0.15
E185634	0.8 ±0.11	0.99 ±0.03	0.95 ±0.11	0.54 ±0.2	0.9 ±0.14	0.86 ±0.05
EAT2	0.66 ±0.2	0.94 ±0.05	0.85 ±0.04	0.63 ±0.09	0.83 ±0.1	0.85 ±0.11
FER^†^	0.92 ±0.06	0.85 ±0.14	–	–	0.95 ±0.05	0.69 ±0.12
FES^†^	0.92 ±0.08	0.82 ±0.11	–	–	–	–
FGR	0.54 ±0.05	0.86 ±0.09	0.78 ±0.13	0.71 ±0.05	0.64 ±0.15	0.85 ±0.09
FRK	0.42 ±0.33	0.96 ±0.04	0.72 ±0.3	0.66 ±0.18	0.65 ±0.25	0.86 ±0.07
GRAP2	0.93 ±0.08	0.97 ±0.03	0.9 ±0.07	0.94 ±0.06	0.95 ±0.08	0.96 ±0.04
GRB10	0.49 ±0.1	0.85 ±0.03	0.85 ±0.05	0.29 ±0.12	0.94 ±0.09	0.43 ±0.16
GRB14	0.48 ±0.23	0.9 ±0.03	0.84 ±0.1	0.47 ±0.11	0.6 ±0.18	0.7 ±0.13
GRB2	0.87 ±0.05	0.91 ±0.06	0.91 ±0	0.91 ±0.06	0.93 ±0.04	0.9 ±0.06
HCK	0.55 ±0.25	0.91 ±0.04	0.82 ±0.13	0.5 ±0.09	0.79 ±0.21	0.75 ±0.08
INPPL1	0.64 ±0.06	0.82 ±0.07	0.84 ±0.15	0.45 ±0.07	0.69 ±0.16	0.8 ±0.07
ITK	0.71 ±0.22	0.85 ±0.06	0.91 ±0.1	0.53 ±0.09	0.95 ±0.06	0.72 ±0.11
LCK	0.55 ±0.09	0.87 ±0.07	0.88 ±0.05	0.5 ±0.07	0.7 ±0.09	0.73 ±0.08
LCP2	0.85 ±0.04	0.76 ±0.07	–	–	–	–
LYN	0.62 ±0.17	0.83 ±0.13	0.75 ±0.16	0.47 ±0.17	0.77 ±0.12	0.67 ±0.18
MATK	0.83 ±0.17	0.79 ±0.07	–	–	–	–
MIST	0.3 0.45	0.94 ±0.04	0.9 ±0.22	0.41 ±0.1	0.5 ±0.5	0.77 ±0.07
NCK1	0.63 ±0.11	0.83 ±0.08	0.78 ±0.09	0.51 ±0.17	0.84 ±0.14	0.71 ±0.13
NCK2	0.71 ±0.14	0.86 ±0.1	0.94 ±0.06	0.39 ±0.07	0.96 ±0.06	0.63 ±0.09
PTK6	0.52 ±0.14	0.89 ±0.09	0.93 ±0.07	0.42 ±0.09	0.78 ±0.19	0.68 ±0.1
SH2B	0.51 ±0.25	0.86 ±0.02	0.85 ±0.05	0.59 ±0.1	0.67 ±0.19	0.78 ±0.06
SH2D1A	0.4 ±0.09	0.88 ±0.06	0.68 ±0.12	0.55 ±0.06	0.63 ±0.21	0.66 ±0.08
SH2D2A	0.47 ±0.11	0.87 ±0.08	0.82 ±0.11	0.43 ±0.13	0.73 ±0.18	0.61 ±0.1
SH2D3C^†^	0.61 ±0.21	0.9 ±0.04	–	–	–	–
SHC1	0.53 ±0.19	0.83 ±0.05	0.92 ±0.04	0.42 ±0.28	0.69 ±0.17	0.71 ±0.12
SHC3^†^	0.71 ±0.04	0.79 ±0.08	–	–	–	–
SOCS2	0.45 ±0.27	0.96 ±0.04	0.9 ±0.14	0.52 ±0.1	0.7 ±0.21	0.89 ±0.1
SOCS5	0.6 ±0.42	0.99 ±0.03	0.8 ±0.27	0.51 ±0.17	0.9 ±0.22	0.84 ±0.12
SRC	0.35 ±0.16	0.95 ±0.03	0.85 ±0.16	0.61 ±0.07	0.65 ±0.21	0.73 ±0.08
TEC	0.57 ±0.11	0.9 ±0.09	0.8 ±0.1	0.53 ±0.13	0.72 ±0.08	0.76 ±0.11
TENC1	0.55 ±0.23	0.89 ±0.08	0.85 ±0.08	0.44 ±0.12	0.8 ±0.12	0.66 ±0.07
TENS1	0.58 ±0.23	0.87 ±0.09	0.87 ±0.05	0.49 ±0.12	0.77 ±0.15	0.78 ±0.11
TNS	0.57 ±0.12	0.87 ±0.05	0.73 ±0.13	0.68 ±0.03	0.7 ±0.09	0.83 ±0.04
TXK	0.47 ±0.1	0.86 ±0.07	0.82 ±0.09	0.53 ±0.17	0.65 ±0.12	0.74 ±0.11
VAV1^†^	0.86 ±0.12	0.88 ±0.04	–	–	–	–
VAV2^†^	0.82 ±0.11	0.83 ±0.14	–	–	–	–
YES1	0.53 ±0.22	0.83 ±0.05	0.75 ±0.2	0.43 ±0.07	0.73 ±0.21	0.69 ±0.12
Avg*	0.57	0.88	0.85	0.53	0.77	0.74

We compare the sensitivity and specificity of each SH2 domain, achieved by using three different datasets (original imbalanced dataset, balanced dataset with randomly chosen negative data and balanced dataset with good negative data derived by self training process).

* The average is computed over all domains except domains indicated with †. The table indicates the datasets generated by the self training strategy perform better.

To assess the importance of the correlation between the amino acid positions we also compared the predictive performance of a linear v.s. a non-linear (i.e. polynomial with degree 2) kernel. In 42/51 = 82.3% cases the polynomial kernel outperfomed the linear kernel according to the AUC ROC measure, which increases to 47/51 = 92.2% cases when we consider the AUC PR measure (see Table S2).

#### Performance comparison

We compare our results with two state-of-the-art tools: SMALI [21], and an energy model approach [31]. We apply these tools as well as our approach to all 51 test sets (SMALI could be applied to 45 test sets as it doesn’t have model for the other 6 SH2 domains). Our model achieves an average AUC ROC of 0.83 and average AUC PR of 0.93 (see Figure 2), outperforming the other two approaches: SMALI achieves AUC ROC of 0.71 and AUC PR of 0.87; the energy model achieves AUC ROC of 0.62 and AUC PR of 0.81. Detailed information on the AUC ROC and AUC PR for each SH2 domain is available in Figure S2 and Figure S3, respectively.

We note that SMALI achieves a very high specificity (0.95 on average) in all 45 SH2 domains when the proposed threshold is used (i.e. relative SMALI score 1), however this comes at the expenses of a very poor sensitivity (0.26 on average). See Table 2 for details.

**Table 2:** Comparison of sensitivity with fixed specificity.

Domains	Specificity	SMALI	Energy-model	SVM-model
		Sensitivity	Sensitivity	Sensitivity
ABL1	0.95455	0.21023	0.03409	0.29545
ABL2	0.95238	0.07500	0.02500	0.55000
APS	1.00000	0.15441	0.10294	0.41176
BCAR3	0.96226	0.05435	0.10870	0.28261
BLK	0.90000	0.26271	0.36441	0.52966
BMX	1.00000	0.11250	0.01250	0.06250
BRDG1	1.00000	0.00000	0.01176	0.40000
BTK	0.96491	0.10680	0.03883	0.36893
CRKL	1.00000	0.26718	0.08228	0.57595
CRK	1.00000	0.37975	0.00000	0.64122
CTEN	0.87500	0.53191	0.17021	0.74468
E105251	1.00000	0.04965	0.04255	0.17021
E109111	0.98246	0.00000	0.05941	0.40594
E185634	1.00000	0.27778	0.09722	0.66667
EAT2	0.96610	0.31429	0.05000	0.37857
FER	0.98333	0.56410	0.02564	0.51282
FES	0.88333	0.67273	0.29091	0.87273
FGR	0.88000	0.32117	0.49270	0.52920
FRK	0.94444	0.21212	0.17803	0.20455
GRAP2	0.88136	0.96914	0.61111	0.96914
GRB10	0.98113	0.13889	0.04167	0.38889
GRB14	0.87931	0.28415	0.22951	0.49180
GRB2	0.88889	0.90476	0.80952	0.90476
HCK	0.89474	0.28241	0.32870	0.51389
INPPL1	0.98361	0.12295	0.04918	0.34426
ITK	0.88372	0.30667	0.73333	0.78667
LCK	0.96429	0.23256	0.09302	0.43256
LCP2†	0.96721	–	0.01695	0.57627
LYN	1.00000	0.11966	0.02564	0.01961
MATK	0.95000	0.11321	0.13208	0.52830
MIST†	1.00000	–	0.19277	0.55422
NCK1	0.94118	0.50459	0.29358	0.44037
NCK2	0.97917	0.31683	0.04950	0.55446
PTK6	0.96667	0.33824	0.00980	0.26961
SH2B	0.96364	0.02198	0.11538	0.42308
SH2D1A	0.92982	0.19162	0.07784	0.22754
SH2D2A	0.88333	0.33036	0.17857	0.47321
SH2D3C†	0.88889	–	0.17105	0.65789
SHC1	0.98039	0.24000	0.07333	0.36000
SHC3	1.00000	0.15517	0.13793	0.12069
SOCS2†	1.00000	–	0.06250	0.39583
SOCS5†	1.00000	–	0.18571	0.75714
SRC	0.97500	0.23476	0.16159	0.27744
TEC	0.95918	0.19018	0.28834	0.24540
TENC1	1.00000	0.13990	0.02073	0.24870
TENS1†	1.00000	–	0.00813	0.14634
TNS	0.94643	0.24876	0.07463	0.49254
TXK	0.94545	0.16541	0.14286	0.43609
VAV1	0.87500	0.35593	0.33898	0.88136
VAV2	0.93878	0.22500	0.15000	0.62500
YES1	0.97500	0.21101	0.08257	0.41284
Avg.*	0.95	0.26	0.17	0.45

We compare the sensitivity of three different approaches. The specificities generated by SMALI program and then we used the same specificities to find the correspondence sensitivity.

† SMALI doesn’t have model for these SH2 domains, therefore, we used high specificity for those domains.

* The average is computed over all domains except domains indicated with †.

In order to directly compare the sensitivities, we identified the threshold for our model so to achieve the same specificity as SMALI (and another threshold for the energy model). The advantage of our approach is evident in this setting too, achieving a sensitivity of 0.45 on average against 0.26 for SMALI and 0.17 for the energy model.

#### Comparison on validated data

Here we test our approach with SMALI on a manually curated and reliable database of SH2-peptide interactions called PhosphoELM [36]. We couldn’t test energy model, since there is no specific threshold that can determine the class.

**Figure 3:**
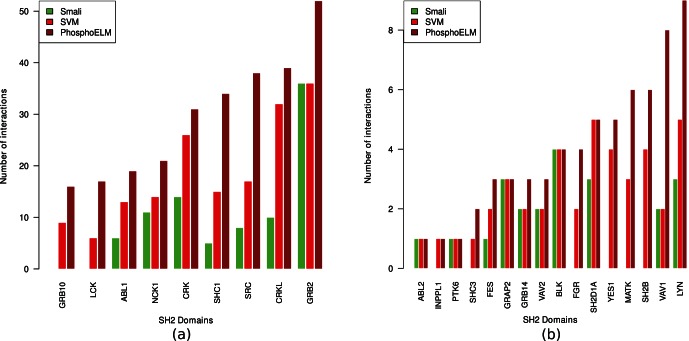
Performance evaluation on manually curated database, PhosphoELM. (a,b) Performance of SMALI and our program on the experimentally validated data. In both (a and b) case the brown bars indicate the actual experimentally validated interactions for individual SH2 domains where the red and green bars indicate the predicted interactions by SVM models and SMALI respectively. (a) Showing those SH2 domains having at least 10 interactions in PhosphoELM 9.0 and (b) Showing the SH2 domains having less than 10 interactions in PhosphoELM 9.0 database.

On this dataset the performance of SMALI (comparable to Scansite [19] although with better accuracy for some SH2 domains) is 112 correct interactions predicted over a total of 335 interactions (26 domains, SMALI doesn’t have models for LCP2 and SOCS2 domains), while our approach identifies 213 true interactions (see Figure 3). In particular, we correctly predicted all the interactions predicted by the SMALI except two interactions for NCK1 and SRC SH2 domain each.

Note that we have taken care to exclude all the interaction data in the PhosphoELM database from our training sets (unfortunately this cannot be done for the SMALI tool since we could use only the pre-trained version).

### Analysis of Existing Approaches

We further investigate the reliability and the generalization capacity of the two state-of-the-art methods: SMALI and energy model.

#### SMALI performance on Microarray data

We use dataset II and III to analyze the correlation between the experimental affinity values and the relative SMALI scores. Dataset II contains 3255 interactions between 105 SH2 domains and 31 pY peptides. The strenght of the interaction is measured by the apparent dissociation constant [34], denoted as K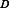. K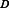 values are available also for dataset III (which contains 3485 interactions between 85 SH2 domains and 41 pY peptides). Interactions are considered reliable when their associated K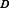 values are lower than 2 µm.

We compute the relative SMALI score for the SH2-peptide interactions in both dataset II and III. A relative SMALI score ≥ 1 is considered indicative of a true interaction.

**Figure 4:**
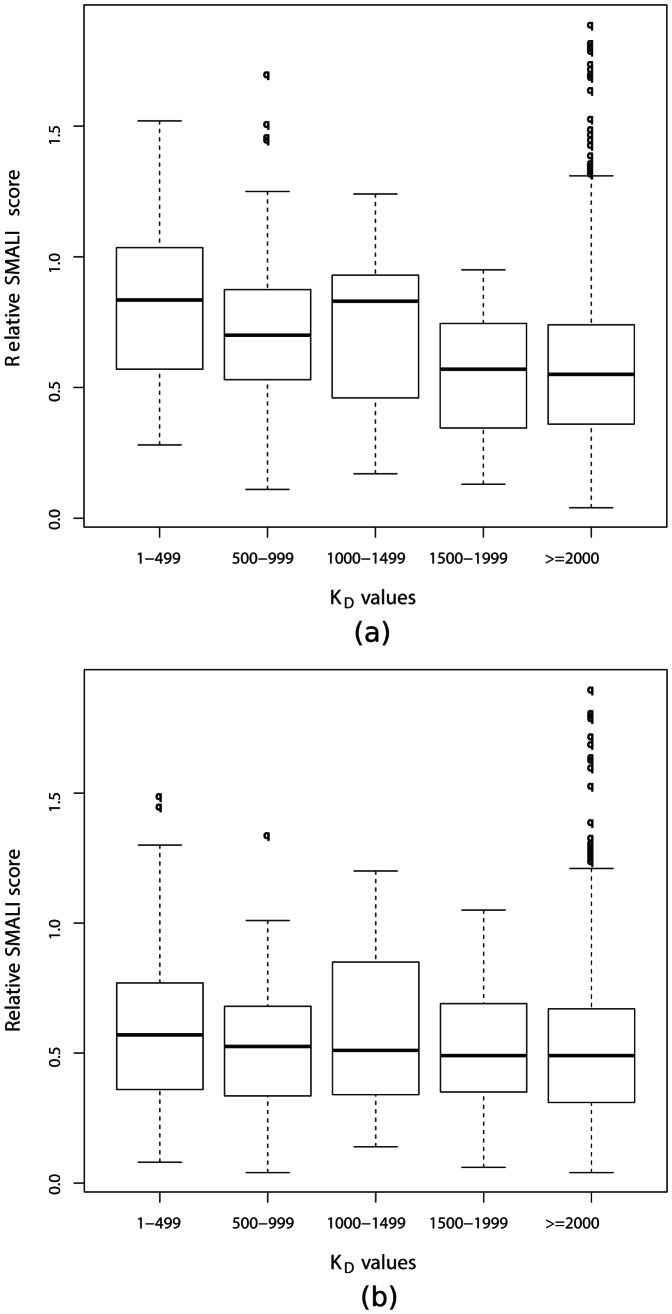
Comparison of the relative SMALI scores with two different microarray experiments. (a,b) Barplots of relative SMALI score with microarray experiments. It separates the K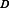 (apparent equilibrium disassociation constant) into five parts, i.e 1–499, 500–999, 1000–1499, 1500–1999 and > = 2000 (unit is in nm). Among them K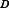 values less than 2000 nm were considered as positive interactions and considered as negative interactions otherwise. (a) Barplot of relative SMALI score with dataset II and (b) Barplot of relative SMALI score with dataset III. In both cases it is clearly observed that there is no correlation between the relative SMALI score with the K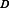 values.

In Figure 4, we report a box plot for the distribution of the relative SMALI scores vs. the K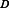 values. We note that a large fraction of interactions that have K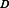 values lower than 2 µm (experimental evidence for a strong binding case) have also low relative SMALI scores (no predicted interaction). If we consider only the non binding interactions we observe a Spearman rank correlation 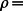 −0.12 w.r.t. the SMALI score (we would expect a large negative value for good predictive capacity). If we consider the binding interactions we see that the average SMALI score is 0.53 ± 0.27, significantly below the unit threshold.

An illustrative case [34] is the interaction between domain ABL1 and peptide ErbB2 pY1139 which has an experimentally K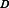 value of 0.16 µm (indicating a very high affinity and a high probability of binding). Here however, the SMALI tool predicts no interaction, giving a relative score of 0.84 (below the unit threshold). Our model instead correctly predicts the binding with a margin of 0.999.

#### Energy model performance on microarray data

The energy model [31] was tuned using information from a large scale microarray experiment [34] (our dataset II).

**Figure 5:**
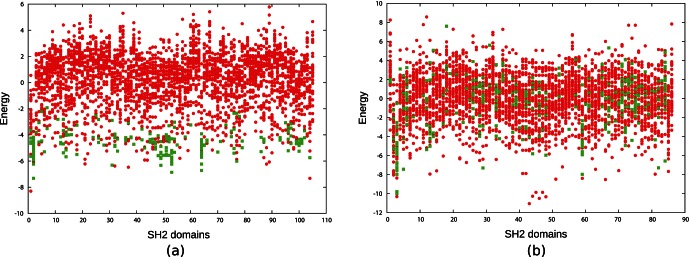
Binding and non-binding energy comparison with different microarray data. (a) Plots for the binding and non-binding energies derived from dataset II, indicates there are clear difference between the binding (red dots) and non-binding interactions (green boxes).(b) With the data derived from dataset III, surprisingly we observed that there is no clear differences between the binding (red dots) and non-binding (green boxes) interactions. The Energy calculation program was kindly provided by Zeba Wunderlich [31].

When we apply this energy model on the dataset II, not surprisingly, we obtain the results reported by [31]; namely TPR 0.90 and FPR of 0.06. More precisely we could determine the threshold value that achieves the reported classification results. In Figure 5 (a) there is a clear energy difference between the binding and the non-binding pairs. The software was kindly made available to us by Zeba Wunderlich.

However, when we apply the same energy model on dataset III [35], we obtain quite a different result. Figure 5 (b) clearly indicates that there are no prominent energy differences between the binding and non-binding pairs. Moreover, we observed in this case there is no threshold that can significantly discriminate between the binding and the non-binding cases (see also AUC ROC results in Figure S4). This seems to indicate an overtraining issue with consequent inability of generalization to a different experimental setup.

### Genome-wide Analysis of Human SH2 Domains

We have performed a genome-wide analysis to uncover unknown interacting partners for each of the SH2 domain used in our study. In this analysis we have made use of prior domain knowledge to remove peptides that are not likely to interact. Specifically, we have considered three criteria for eligibility of a given pair peptide-domain: 1) presence of the tyrosine (Tyr) residue in the peptide, 2) experimentally verified phosphorylation of the tyrosine in the peptide, 3) co-cellular localization of the mature protein that contains the peptide and the protein that expresses the domain.

We have extracted the set of peptides from the UniProtKB/Swiss-Prot database [37], which is a well known manually curated and reviewed database. At the moment of the analysis, the UniProtKB/Swiss-Prot database, release 2012-06, contained 20225 human proteins with ∼300 000 (298 637) tyrosine containing peptides.

The second filter has been implemented using the annotated information in the PhosphoSitePlus database [38]; in this way we have selected only those phosphotyrosine peptides whose phosphorylation has been experimentally verified. At the moment of the analysis the PhosphoSitePlus database contained 30228 phosphorylation sites from 10688 human proteins. We have ignored those peptides that were not present in the UniProtKB/Swiss-Prot database obtaining finally 27481 phosphorylation peptides out of 9621 proteins.

The third filter was implemented considering the terms relative to the sub-cellular localization hierarchy in the controlled vocabulary of the Gene Ontology database [39]. In case of multiple cellular locations (e.g. GRB2 protein can be found in nucleus, cytoplasm, endosome and golgi apparatus [40]) we consider a peptide viable for interaction if it shares at least one of the terms with the domain. Finally, we ignored proteins (such as SHD/E105251) for which no localization annotation is available.

All eligible peptides-SH2 domains pairs were scored by the trained models and ranked according to the SVM scores. Considering the top ranked and most reliable 50 predictions (see File S1), we offer the following hypothesis.

1. The SH2-domain of ABL1 is predicted to bind to Y307 of the adaptor protein GAB1. ABL1 is part of the oncogenic protein BCR-ABL, which is generated by a (9;22) translocation resulting in the so-called Philadelphia chromosome and is found in CML (chronic myelogenous leukemia) [41]. BCR-ABL has been shown to be dependent on GAB adaptor proteins, in particular GAB2. It has been demonstrated that GAB2 in CML cells confers resistance to multiple BCR-ABL inhibitors [42]. The known interaction between BCR-ABL on one side and GAB adaptor proteins on the other side can be described as following: the small adaptor protein GRB2 binds to phosphorylated Y177 on BCR-ABL via its central SH2-domain and via its SH3-domains it interacts with proline-rich sequences within both GAB proteins, GAB1 and GAB2 [43]. Our finding would suggest a second so far unknown mode of BCR-ABL/GAB1 interaction that is GRB2-independent and based on a direct interaction between the BCR-ABL (ABL1) SH2-domain and tyrosine-phosphorylated GAB1.
2. Our model indicates that the SH2-domain of the adaptor protein CRKL interacts with phosphorylated Y215 of ABL1. Interestingly, CRKL has been found to be one of the predominant substrate of the oncogenic kinase BCR-ABL [44]. This suggests that CRKL is not only a substrate, but also an interaction partner of BCR-ABL. Most likely, the interaction promotes phosphorylation.
3. TEC-family kinases are multidomain cytoplasmic tyrosine kinases, which comprise, amongst others, an N-terminal PH-domain. This PH-domain interacts with the phospholipid phosphatidylinositol-3,4,5-trisphosphate (PIP3), which is generated by PI3K enzymes upon receptor activation [45]. PI3K class IA, which is activated downstream of multiple receptors, such as immune receptors and cytokine receptors, comprises various catalytic and regulatory subunits [46]. Interestingly, our model found that different TEC-family kinases (BTK, ITK, and TEC) via their SH2-domains can interact with various regulators subunits of PI3K class IA: BTK interacts with Y74 of p85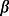; ITK interacts with Y464 of p85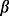, with Y467 and Y556 of p85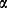, and with Y199 of p55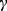; TEC interacts with Y74 of p85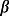 and Y556 of p85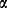. Since the regulatory subunits of PI3K are necessary to guide the catalytic PI3K subunits to their substrate in the plasma membrane, interaction of TEC kinases with the regulatory subunits would enable them to be close to the newly generated PIP3, which then is necessary for their activation. Using such a mechanism, TEC kinases always could be close to newly generated PIP3 enabling immediate activation.
4. The inositol-5-phosphatase SHIP1 has been shown to interact with TEC via TEC SH3-domain binding to a proline-rich sequence in the C-terminus of SHIP1 [47]. Our model suggests that there is a second mode of interaction between SHIP1 and TEC, namely between the SH2-domain of TEC and the phosphorylated Y221 of SHIP1. Such a mode of interaction would be called €bidentate€ and has already been found for the interaction between SHIP1 and one of its main interaction partners, the adaptor protein SHC. In that case, the PTB-domain of SHC binds to a phosphorylated tyrosine within the C-terminus of SHIP1 and the SH2-domain of SHIP1 binds to a phosphorylated tyrosine within SHC [48]. Using such a bidentate mode would clearly strengthen the interaction between the two partners.
5. The inositol-5-phosphatase SHIP1 counteracts PI3K signaling via its centrally located catalytic domain, hydrolyzing the phospholipid PIP3 [48]. Moreover, it has been demonstrated to negatively regulated p21Ras signaling via complex formation with the adaptor protein DOK1 and the p21Ras GTPase activating protein RASGAP [49]. So far, such an interaction or function has not been described for the second family member, SHIP2. Interestingly, our model suggests the interaction of the SH2-domain of SHIP2 (INPPL1) with phosphorylated Y650 of another p21Ras GTPase activating protein, RASA2. This would suggest that both SHIP proteins can realize comparable functions, however, using different modules. The qualitative outcome might be the same, although regulation might be differentially accomplished.
6. Induction and regulation of calcium mobilization downstream of the B-cell antigen receptor is crucial for differentiation and activation of B-lymphocytes. It was shown that the tyrosine-phosphorylated adaptor protein DOK3 interacts with the SH2-domain of the adaptor protein GRB2. Stork et al have demonstrated that this DOK3/GRB2 module negatively influences the assembly of the calcium initiation complex and/or inhibits the enzymatic activity of the tyrosine kinase BTK, which is crucial for calcium mobilization to occur [50]. Our data indicated that the SH2-domain of BTK directly interacts with DOK3 phosphorylated on Y398.Though our analysis was performed in the human system and the study by Stork et al was making use of the chicken DT40 B-cell system, sequence comparison suggests that the same tyrosine (Y398 in human and Y331 in chicken [50]) could bind to GRB2 and BTK. This would add another layer of complexity to the regulation of calcium mobilization in B-lymphocytes.

We performed a second type of analysis on the same top 50 predictions in order to uncover novel functionalities using the DAVID tool [51]. The tool offers the possibility to perform a term-centric enrichment analysis on more than 40 different annotation categories. Analyzing the highly enriched results we found, for example, that CRKL interacts with a group of proteins (Swiss-Prot ID: P42684, Q9UQM7, Q13555, P00519, P42345, Q13554, Q13557) that play an important role in ErbB signaling pathway (as reported in the KEGG pathway database [52]). We note that the SMALI tool misses all these associations (see File S2 for more details).

Finally, we found that some peptides (P05067-755-761 NGYENPT, P61106-12-18 FKYIIIG, P09211-48-54 CLYGQLP, P25788-103-109 FGYNIPL, P29350-562-568 DVYENLH, Q05397-923-929 KVYENVT, P08865-137-143 ASYVNLP, P13533-552-558 KLYDNHL, P56945-10-16 ALYDNVA, O15530-374-380 GNYDNLL) are predicted to interact a-specifically with more than 40 SH2 domains. In addition, we observed 3-phosphoinositide-dependent protein kinase 1 (Uniprot-id: O15530) targeted by the most number (34 domains) of SH2 domains that share the same cellular compartment and functions annotated in GO-term database.

### Conclusions

SH2-peptide interactions are an important component of cell signaling. Because of the limited availability of experimentally proven interactions, machine learning approaches have to be used in order to generalize to combinations that have not been experimentally investigated. High-throughput experimental methods seem to be a perfect data source for training these models. There are, however, two order of problems in these data: a) a significant noise component, and b) quite an imbalance between confirmed interactions (positive data) and experimentally proven non-interactions (negative data). In addition, current state-of-the-art models for SH2-peptide interaction prediction are based on linear models, which are not capable of handling complex interactions patterns.

In this paper, we propose a model that tackles these issues. On the one hand, we propose an iterative re-balancing strategy to compensate the imbalance problem. On the other hand, to model complex interaction patterns we use a polynomial kernel support vector machine and we avoid overfitting issues employing a regularization scheme.

In our study we used three high throughput data: two derived from microarray experiments and one from a peptide array library experiment. We carefully compared our approach with state-of-the-art tools, namely SMALI and an energy based structural model, achieving a significantly better generalization performance (measured as cross validated AUC ROC and AUC PR). This result was additionally confirmed on a manually curated database (PhopshoELM) of experimentally validated SH2-peptide interactions.

Finally, we performed a genome-wide prediction of human SH2-peptide interactions. We report some novel interactions between SH2 domains and tyrosine-phosphorylated proteins: as an example we find that oncogenic protein BCR-ABL (ABL1) may directly bind (not dependent on GRB2) with pY307 of the adaptor protein GAB1.

We have made the learned models, as well as all the genome-wide interaction predictions, available to the community.

## Materials and Methods

### High Density Peptide Arrays Data

#### Dataset I

From the NetPhorest database [53] we collected information on 61 SH2 domains and 920 phosphorylated peptides for a total of 14678 interactions. After removing all redundancies we obtained 7544 positive interactions.

Note that for high density peptide array experiments, there is evidence only for positive interactions. One cannot however assume that the remaining 61×920−7544 = 48576 interactions are of the non-binding type (i.e. negative interactions). It can happen that these domain-peptide interactions were just not observed in the assay due to the experimental stringency (i.e. consistency among replicates).

### Microarray Data

#### Dataset II

From the protein microarray experiments in [34] we have considered the SH2-peptide interactions data excluding the PTB-peptide interactions. There are 115 SH2 domains and 20 singly phosphorylated peptides from ErbB2 and ErbB3 proteins. Note that there are 10 cases where a single protein has both a C-terminal and N-terminal SH2 domain. Since the database does not report the assignment of which peptide specifically binds to which of the two domains (N and C terminal) we have discarded the interactions related to these proteins. From this dataset we have collected 105×20 = 2100 interactions, with 160 positive interactions and the remaining 2100−160 = 1940 being considered as negative interactions.

#### Dataset III

From the protein microarray experiments in [35] we have considered the SH2-peptide interactions data excluding the PTB-peptide interactions. In this study there are 85 SH2 domains and 41 singly phosphorylated peptides from EGFR, FGFR, IG1FR proteins. We have proceeded in an analogous fashion as with dataset II and we have collected 85×41 = 3485 interactions with 314 positive interactions and 3485−314 = 3171 negative interactions.

### Curated Test Data

#### Dataset IV

From PhosphoELM [36], which is a high-quality manually curated database, we have extracted the interactions for 28 SH2 domains with 339 peptides.

### Dataset Compilation

We have combined positive and negative data from two microarray datasets (dataset II and dataset III) using the measured apparent equilibrium dissociation constants [34], [35] (K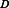 value) to determine the class label. SH2-peptides interactions with K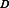 values lower than 2000 nM were considered as binding (positive interactions) while all other pairs were considered as non-binding (negative interactions).

The total number of positive interactions is 474 (160 and 314 respectively from dataset II and dataset III), while the total number of negatives interactions is 5111 (2100−160 = 1940 and 3485−314 = 3171 respectively).

Dataset I contains 7544 positive interactions and no negative interactions. Among the 474 positive interactions in dataset II and III, 247 (112 and 135) were in common between the microarray and the peptide array data. After removing the positive interactions of dataset I from dataset II and III, we obtain 227 (48 and 179) unique positive interactions for dataset II and III.

Surprisingly, we found 149 interactions for which the microarray data and the peptide array data are in disagreement, i.e. it is positive for dataset I but negative for dataset II and III. We have therefore discarded those interactions to reduce unreliable and conflicting information in the training phase. As a consequence the number of negatives from dataset II and III is reduced to 5111−149 = 4962, and the number of positives in dataset I is reduced to 7544−149 = 7395.

To compose our datasets we used the positive interactions from the more reliable dataset I (7395) and the available negative interactions from dataset II and III (4962). The non redundant positive data derived from microarray experiments was kept for validation purposes.

For each of the 61 SH2 domain in dataset I we compile a separate dataset. We discard 10 domains that have less than 40 positive interactions since no complex model can be reliably fit.

Finally we have 61−10 = 51 SH2 domains for which we have 6742 positive and 2523 negative interactions. See Table 3 for further details.

**Table 3:** Ensemble data from literature and the final data used in this study after compilation.

	Original Data						Selected Data					
Datasource	# D	# P	# I	#Pos	#Neg	#Ukn	#D	#P	#I	#Pos	#Neg	#Ukn
Dataset I	61	920	56120	7544	–	48576	51	880	44800	6742	–	38138
Dataset II	105	20	2100	160	1940	–	51	20	1020	48	851	–
Dataset III	85	41	3485	314	3171	–	46	41	1886	179	1672	–
Dataset IV	63	359	–	878	–	–	28	197	–	339	–	–

# D is the number of domains, # P is the number of peptides, # I is the number of interactions, # Pos is the number of positive data, # Neg is the number of negative data and # Ukn is the number of unknown data.

### Data Modeling

Previous studies show that residues in the close vicinity of the phosphotyrosine are highly predictive for domain-peptide binding [19], [21], [31]. For example it is known that the SH2 domain of CRK binds peptides where amino acid Leu or Pro is in position +3, however the presence of other amino acids (i.e. His, Arg, Ala, Pro) in position +1 and +2 can inhibit the interaction. [32].

Here we follow the literature and restrict the peptide sequence to 6 specific positions, namely we extract the amino acids in positions ranging from 2 upstream to 4 downstream of the phosphotyrosine residue. A peptide is therefore mapped into a binary vector 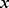 living in a 20×6 = 120 dimensional (the central amino acid is always a phosphotyrosine and is therefore not included in the encoding), that is, for each position, we reserve 20 dimensions (one for each amino acid) and encode the amino acid type with a 1 in the corresponding dimension and 0 elsewhere.

For each domain 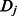 we compile a data set encoded as a set of pairs (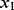,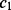),.,(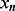,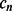) where, 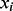 is the binary feature vector for peptide 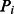 with the class label 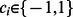. The class label is +1 if the domain 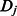 interacts with peptide 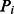 and −1 otherwise.

### Predictive Model

As predictive model we employed a regularized polynomial kernel support vector machine SVM [54]. We used the SVM implementation in C language provided in SVM 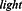 [55].

#### Predictive performance measures

We formulated a learning problem for each SH2 domain. The predictive performance for each problem was assessed computing 5 measures: sensitivity, specificity, precision, area under the receiver operating characteristics curve and area under the precision recall curve. These are defined as: 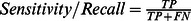, 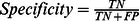, 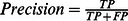, where TP denotes true positive, that is SH2 domain-peptide pairs predicted correctly as binding pairs, TN denotes true negative, i.e. SH2 domain-peptide pairs predicted correctly as non-binding pairs, FP denotes false positive, i.e. SH2 domain-peptide pairs predicted incorrectly as binding pairs and FN denotes false negative, i.e. SH2 domain-peptide pairs predicted incorrectly as non-binding pairs.

The area under the receiver operating characteristics curve (AUC ROC) is defined as the area under the curve obtained by plotting the fraction of true positives out of the positives (TPR = true positive rate) vs. the fraction of false positives out of the negatives (FPR = false positive rate), at various threshold settings.

The area under the precision recall curve (AUC PR) is defined as the area under the curve obtained by plotting precision as a function of recall.

#### Model fitting protocol

The model parameters that can be tuned are the polynomial degree 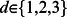 and the cost parameter 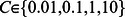 used to trade off generalization for data fitting.

In order to estimate the expected predictive performance for our approach we computed the 5 measures described above under a stratified 5-fold cross-validation scheme.

In particular, all the available data is partitioned into 5 parts ensuring the same proportional distribution of positive and negative instances in each part. Each part is used in turn as a held out test set, while the remaining 4 parts are used as training set. We determined the optimal parameters configuration (i.e. the pair 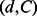) as the minimizers of a 10-fold cross-validated AUC ROC measure for each of the 5 training sets, independently. We then selected the most frequent parameters configuration pair 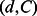. This was the configuration finally used in the stratified 5 fold cross-validation.

We also performed 10 repetitions of a 75%, 25% random split of the available data to create 10 train/test data sets. We proceeded in an analogous fashion (10-fold cross-validation) to determine the most frequent parameters configuration pair 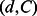. The final average performance estimate is comparable to that obtained in the 5-fold cross-validation setting (see Figure S1).

### Machine Learning Model

#### Modeling issues: a short review on the imbalanced dataset problem

From an in-silico modeling point of view, a key characteristic of the problem at hand is that the available supervised information on peptide binding induces imbalanced datasets, i.e. for certain SH2 domains, information on real interactions can be up to 15 times more abundant than information on the lack of interaction (see Table S1). In literature it is known (see [56] for a recent survey) that severe imbalanced class distributions negatively affects the performance of machine learning approaches. The exponential increase in the number of publications dedicated to imbalanced data management in the last decade is a clear indication of the importance of the issue.

The problem arises since mainstream machine learning algorithms are not designed to compensate for skewed class distributions, and concentrate on being accurate only on the majority class. Two major causes of problems with class imbalance are: a) the choice of an adequate performance measure to guide the selection of the best hypothesis, and b) the discrepancy in the data distribution between the model induction (train) and the model application (test) phase [57].

To illustrate point a) consider a typical protein interaction prediction problem: while the number of possible interactions grows quadratically with the number of proteins, the number of positive interactions grows typically only linearly (i.e. one protein will bind to a small fixed number of other proteins). In this case the standard accuracy measure is not appropriate since a rational choice based on maximizing the predicted accuracy (in an equal cost scenario) would inevitably be biased towards the majority case, and hence the algorithm will almost always predict a negative/no-interaction response. To deal with this issue, there have been developed techniques that try to explicitly and differently model the cost of each type of mistake. A major drawback of this approach is that the optimal cost matrix is unknown and the result is therefore, highly dependent on expert knowledge and a set of arbitrary/heuristic choices.

As for point b), it has been recognized that the issue is linked to the *within-class imbalance* problem and the *small disjuncts* problem [58]. The phenomenon arises when the class concept is composed by many sub-concepts/sub-clusters each represented by relatively few examples. Standard approaches achieve suboptimal results here, since not enough examples are available to model an adequate response for these exceptional although significant cases.

Standard approaches are further compromised if the sampling procedure in the test phase differs from the one used to collect the training set. This typically happens when a small sub-cluster in the training set is over-represented in the test set (e.g. if cellular conditions or experimental parameters changes during data collection).

Some guidelines are however emerging in the machine learning literature on how to counter-balance the small-disjunct problem; the main recommendation is to prefer intelligent over-sampling techniques to down-sampling as the latter always implies a loss of information which ultimately results in under-performing models. General approaches to over-sampling (such as the popular SMOTE (synthetic minority oversampling technique) [59]) have the drawback of requiring an explicit instance representation (generally in some vector space of relatively low dimensionality) and are therefore more difficult to adapt to the type of data typically encountered in bioinformatics applications (i.e. sequences or graphs). Fortunately, in our case we can circumvent this problem by exploiting a useful property of the datasets we have at our disposal: instead of creating novel instances we can make use of a large quantity of results available from high density peptide array experiments; specifically we can select those peptides for which no definitive interaction information is available. In this way, we do not have to invent plausible biological peptide sequences to populate the neighborhood of minority class representatives. Rather, we have to perform the easier task of estimating when an existing peptide is likely to belong to the minority concept.

#### Learning issues: a short review on the semi-supervised problem

The task of estimating when an existing peptide belongs to the non-interaction class can be viewed as a special instance of the well studied semi-supervised learning task (SSL) [60], i.e. learning from a small amount of labeled data and a large amount of unlabeled data. Here, differently from the general problem formulation, we are interested in using the unsupervised material to have a better characterization only of the minority class; in our case, the one representing the absence of protein-peptide interaction.

Several strategies have been developed to deal with the SSL problem, such as self-training, expectation maximization (EM) with generative mixture models, co-training, transductive support vector machines, and graph-based methods. In order for SSL methods to use effectively the small amount of labeled data, strong model assumptions need to be made. Note that this is a critical step, as it has been observed that if the model assumptions are not matching the problem nature, then using unsupervised material hurts the predictive performance. We therefore review the assumptions made by each SSL strategy, matching them to our specific application case.

Expectation maximization techniques with generative mixture models can be used when data is well clustered according to the class information. In our case, clustering peptides using a metric that makes use of all amino acid information does not induce a good class separation, in fact it is believed that binding is the result of the joint presence of only few specific amino acids in specific positions.

Co-training is used when features naturally split into two sets, with a different instance coverage, but this is not the case for our application.

Graph-based methods perform a type of information spreading on unsupervised instances that is meaningful when two nearby instances (i.e. instances with similar features) tend to be in the same class. For the same reasons detailed for the EM case, this type of bias is not appropriate for our application.

Finally, we resort to the self-training approach, which relies only on the good discriminative properties of the base classifier. The method is a simple wrapper scheme around a base classifier: the initial labeled data is used to train the classifier which then assigns a label to the remaining material. The most confident predictions are then iteratively added to the training set and the classifier is re-trained. The method name derives from the fact that the classifier uses its own predictions to teach itself. The bias is now adequate if the base classifier can learn the importance of each combination of amino acids in specific positions.

#### Regularized non-linear support vector machine

Predictive systems based on PSSMs are essentially linear classifiers. To see why, we review the design principles for the state-of-the-art PSSM system SMALI [21]. Here a procedure is employed to compute a weight matrix 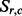 with 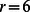 rows and 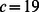 columns (the Cys amino acid is not represented). The peptide-protein interaction is predicted computing a score value as 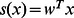, where 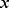 is a 114 dimensional vector constructed as specified in the Data Modeling Section, 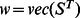 where 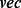 is an operator that transforms a matrix 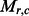 into a column vector 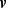 of size 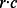, by concatenating all columns. Peptides scoring above a predefined threshold are classified as binding. In SMALI a relative score is defined in such a way as to have a unit threshold. The relative score is then the ratio between the original score and a reference score 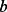. The classifier becomes 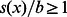 which can be rewritten in a canonical linear form as 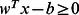.

From a machine learning perspective, the procedure employed in SMALI to compute 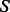 and 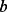 is rather involved and heuristically motivated. The elements in the matrix 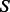 are computed from OPAL [21] experimental results, and essentially correspond to the difference between the average counts of position specific amino acids in the positive examples minus the overall average counts (this corresponds geometrically to find the difference vector between the center of mass of the positive set and the overall set. Had it been the difference vector between the center of mass of the positive set and the center of mass of the negative set, it would have resembled the well known Fisher discriminant model). These quantities are then transformed so to extract information theoretic quantities as a proxy of the importance (the weight) of each position specific amino acid.

The domain specific reference score value 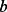 is defined as the value corresponding to the top 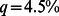 raw SMALI scores over all human proteins in the Swiss-Prot database that contain Tyr. The choice of the fixed value 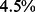 was based on two experiments over the domains BRDG1 SH2 and GRB2 SH2, arbitrarily chosen as representative cases. The optimal (w.r.t. F-measure) threshold for the raw SMALI score was computed using a selection of 1488 peptides for BRDG1 (yielding a SMALI value of 1.4) and 720 peptides for GRB2 (yielding a SMALI value of 1.65). The percentiles corresponding to these thresholds were 3.5% for BRDG1 and 5.5% for GRB2. The final value 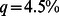 was chosen as their average. As a result of all these choices, it is hard to identify a clear objective for which the proposed linear solution should be optimal.

Here we propose two ways to improve PSSM linear models: 1) upgrading the system from linear to non-linear and 2) making the system more robust using *regularization* techniques.

Non linear models allow to express decision rules that can differentiate between the joint status of two or more position specific amino acids and the status of the same elements taken independently. In this way non additive effects can be modeled, for example consider a case whereby the presence of amino acid Asn in position +2 alone is not sufficient to guarantee the interaction and neither is the presence of amino acid Lys in position −1. However if these amino acids are occurring in their respective positions at the same time then the binding occurs. Another type of non-linear effect could raise when the presence of a either one or the other amino acid is sufficient for binding but when they are both present than they interfere with each other and no binding takes place.

As a non linear model we choose to upgrade the standard linear SVM via a polynomial *kernel* of the type 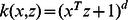. To see how a kernel allows an otherwise linear model to become sensitive to multiple interacting amino acids, we briefly review the ideas behind the “ernel trick” Given a linear predictive model 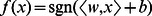, where 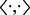 represent the dot product operation, one can employ the support vector machine [6] algorithm to determine the support elements (the non zero 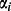 select which, among all 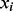, are the support vectors) and rewrite the decision function as 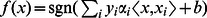. The trick consists now in replacing the standard dot product with a “kernel function”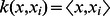, i.e. a function which is symmetric and positive semi-definite [61]. Choosing an appropriate kernel function allows us to transform a linear classifier into a non linear one. Exploiting results known from the Reproducing Kernel Hilbert Spaces theory one can equate the choice of a kernel function to the selection of an appropriate feature mapping function 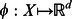 and write 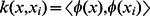. It is often possible to compute efficiently 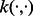 without having to compute 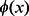, i.e. without having to represent the instances explicitly in the transformed feature space. This is particularly beneficial when the size of representation is very large (it can also be infinite in the case of Gaussian kernels). One of such cases is the polynomial kernel; to fix the ideas we provide the explicit mapping of a quadratic kernel 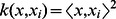 in the simple case of two dimensional instances would result in 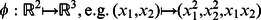,

In our domain this means that with a quadratic kernel we can model interactions between any of two positions in the peptide. Note that in the general case one can account for all interactions of order 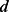 by employing a polynomial kernel of degree 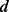, without having to explicitly enumerate all combinations. In our case, with 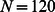 and a polynomial of degree 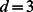, we are implicitly working in a vector space with 300K dimensions. Here, the number of different monomials of degree 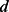 for 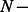
dimensional vectors can be computed as:
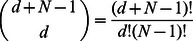



To further improve the predictive performance we propose to use *regularization* techniques to counter balance over-training phenomena, i.e. the tendency to specialize the model on the specific training data idiosyncrasies. It is an unfortunate state of affairs that this aspect is often ignored in the development of novel bioinformatics systems. In practice a regularized predictor is more robust to noise and offers guarantees of a better predictive behavior on unseen instances. Amongst the several ways to ensure a regularized solution, we adopt the strategy championed in SVM, i.e. we minimize the complexity of the model by constraining the size of 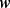 and the degree of the polynomial 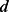. We do this using a cross-validation procedure in order to achieve a good compromise with respect to the training misclassification error. In practice the SVM optimal hyper plane is determined as the solution to a minimization problem where the objective function combines a term proportional to the training error and a term proportional to the complexity of the model (computed as the norm of the hyper plane coefficient vector). The mixing coefficient that weights the importance of the error w.r.t. the model complexity and the degree of the polynomial kernel are selected from a finite set of alternatives. The best parameters combination is chosen by evaluating the predictive performance of each specific model over a held out set of instances (the validation set). Note that the performance of the selected model is evaluated over a further held out set of instances (the test set) that has never been used neither in the training phase nor in the validation phase.

## Supporting Information

Figure S1 **Averaged performance value for rendom train-test splitting method.** Averaged AUC ROC and AUC PR achieved by random train-test splitting method.
(PDF)

Figure S2 **AUC ROC comparison.** AUC ROC comparison of three different methods (SVM, SMALI, Energy model) for each SH2 domain.
(PDF)

Figure S3 **AUC PR comparison.** AUC PR comparison of three different methods (SVM, SMALI, Energy model) for each SH2 domain.
(PDF)

Figure S4 **Binding and non-binding energy comparison.** AUC ROC comparison of binding and non-binding energy for two different microarray data using energy based model.
(PDF)

Table S1 **Imbalanced dataset.** Imbalanced level for confirmed presence or absence of peptide interactions with techniques 51 SH2 domains of this study.
(PDF)

Table S2 **Comparison of linear and non-linear kernel.** AUC ROC and AUC PR comparison of linear and non-linear kernel for each SH2 domain.
(PDF)

File S1 **Genome-wide top predictions by each SH2 domain.** Genome-wide top 50 predicted interactions for each human SH2 domain are reported.
(XLSX)

File S2 **Term-centric singular enrichment analysis using DAVID tool.** Term-centric singular enrichment analysis which identify enriched annotation biological terms associated with the predicted proteins. The smaller *p*-values indicate higher enrichment.
(XLSX)
