# Supplementary material for: Semi-Supervised Prediction of SH2-Peptide Interactions from Imbalanced High-Throughput Data
Source: PLoS One. 2013 May 17;8(5):e62732. doi: 10.1371/journal.pone.0062732 (PMC3656881; doi:10.1371/journal.pone.0062732)

Figure S1: **Averaged AUC ROC and AUC PR achieved by random train-test splitting method:** (a,b) Showing the AUC ROC and AUC PR for the SVM performance, respectively. We achieved AUC ROC 0.9 and AUC PR 0.96.

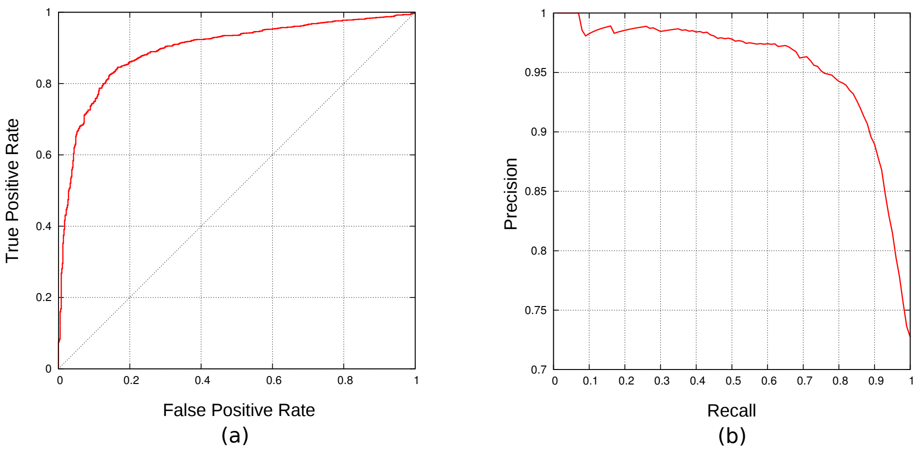

Supplement: Figure S1 — Averaged performance value for rendom train-test splitting method. Averaged AUC ROC and AUC PR achieved by random train-test splitting method. (PDF) [file pone.0062732.s001.pdf]
