# Supplementary material for: Semi-Supervised Prediction of SH2-Peptide Interactions from Imbalanced High-Throughput Data
Source: PLoS One. 2013 May 17;8(5):e62732. doi: 10.1371/journal.pone.0062732 (PMC3656881; doi:10.1371/journal.pone.0062732)
Supplement: Figure S2 — AUC ROC comparison. AUC ROC comparison of three different methods (SVM, SMALI, Energy model) for each SH2 domain. (PDF) [file pone.0062732.s002.pdf]

Figure S2: **AUC ROC achieved by SVM (red lines), SMALI (green dashed lines) and Energy model (blue dotted lines) for each SH2 domain.**

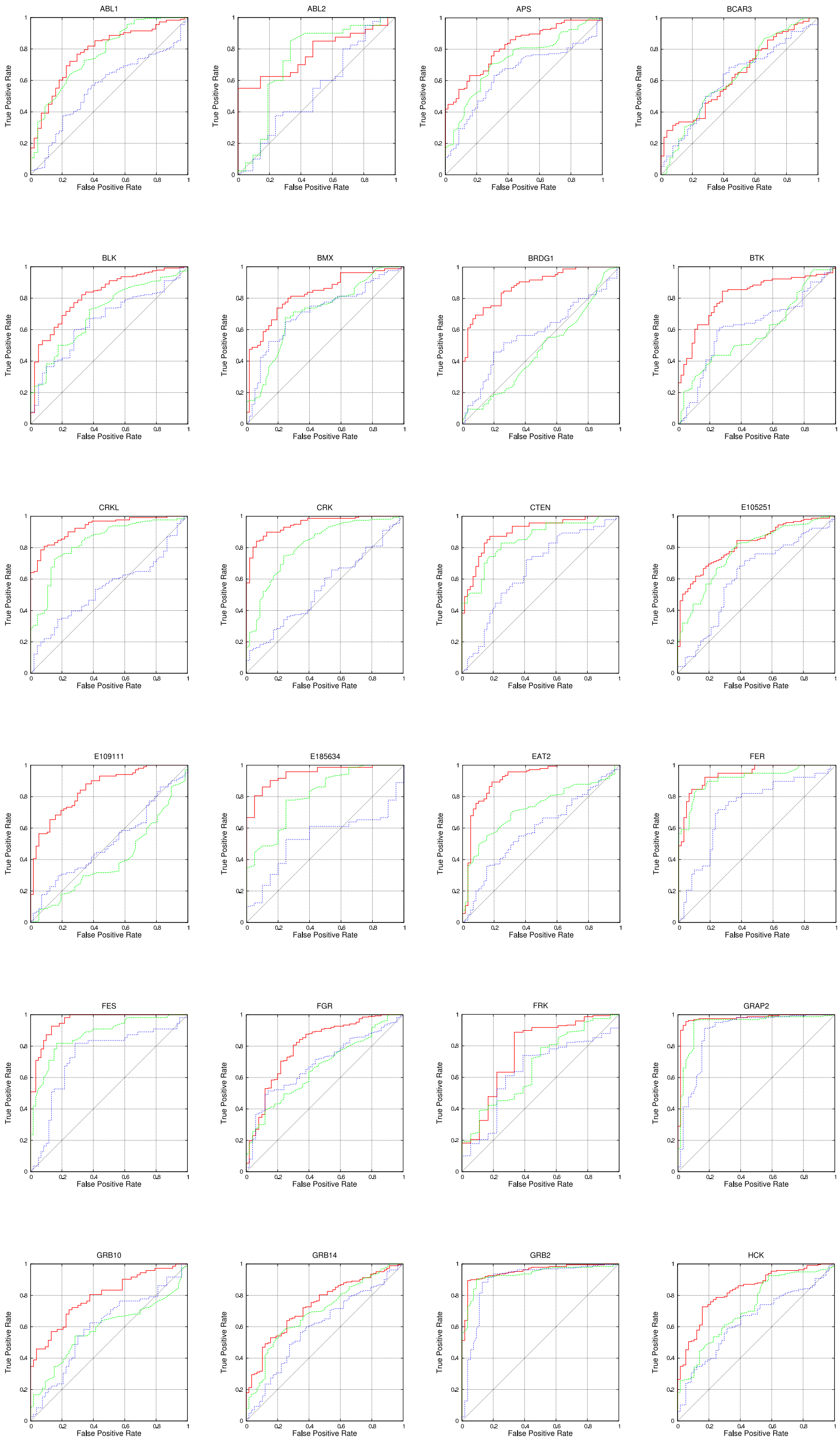

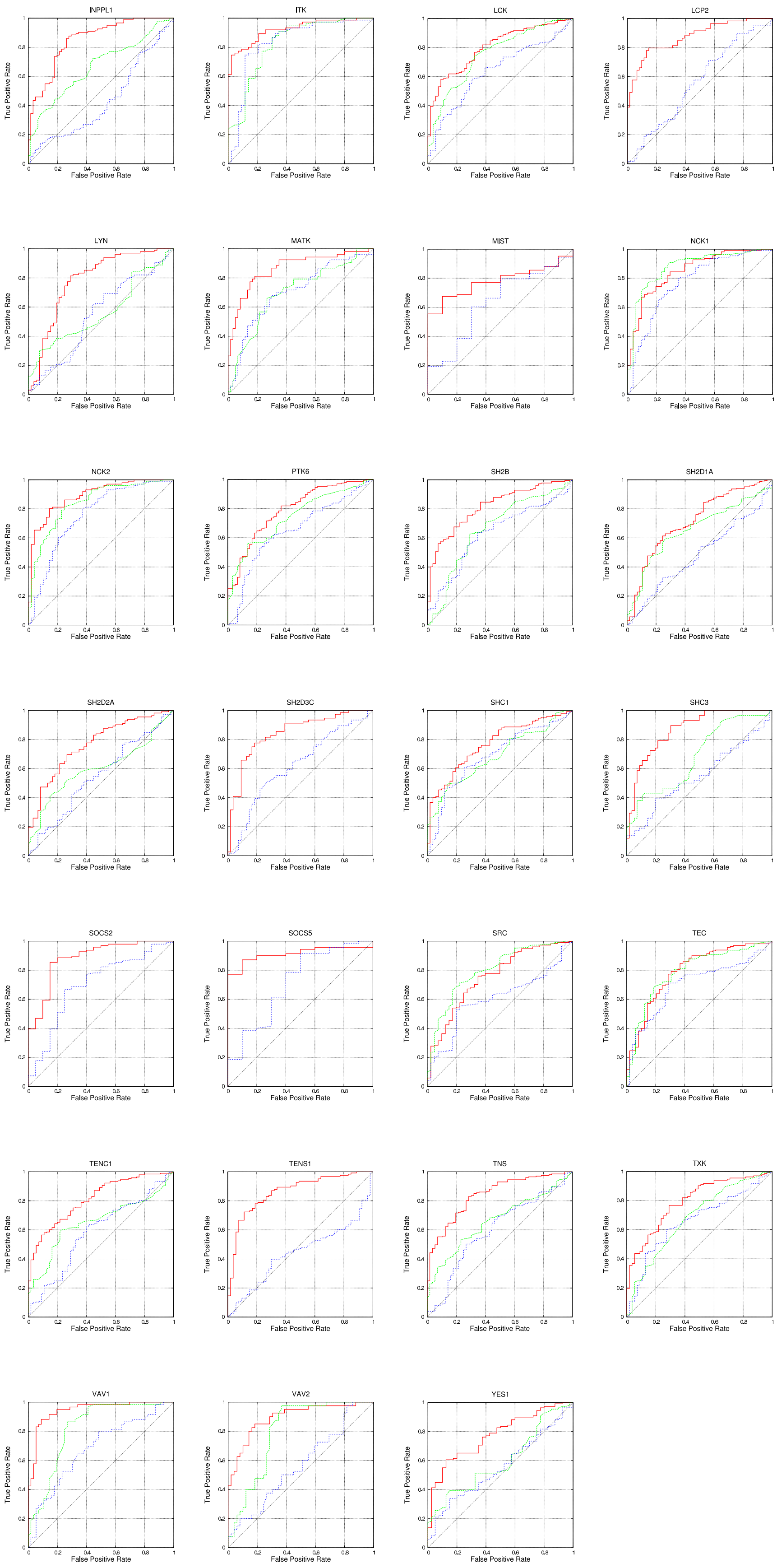

INPPL1
ITK
LCK
LCP2
LYN
MATK
MIST
NCK1
NCK2
PTK6
SH2B
SH2D1A
SH2D2A
SH2D3C
SHC1
SHC3
SOCS2
SOCS5
SRC
TEC
TENC1
TENS1
TNS
TXK
VAV1
VAV2
YES1
True Positive Rate
False Positive Rate
