# Supplementary material for: Semi-Supervised Prediction of SH2-Peptide Interactions from Imbalanced High-Throughput Data
Source: PLoS One. 2013 May 17;8(5):e62732. doi: 10.1371/journal.pone.0062732 (PMC3656881; doi:10.1371/journal.pone.0062732)
Supplement: Figure S3 — AUC PR comparison. AUC PR comparison of three different methods (SVM, SMALI, Energy model) for each SH2 domain. (PDF) [file pone.0062732.s003.pdf]

Figure S3: **PR curve achieved by SVM (red lines), SMALI (green dashed lines ) and Energy model (blue dotted lines) for each SH2 domain.**

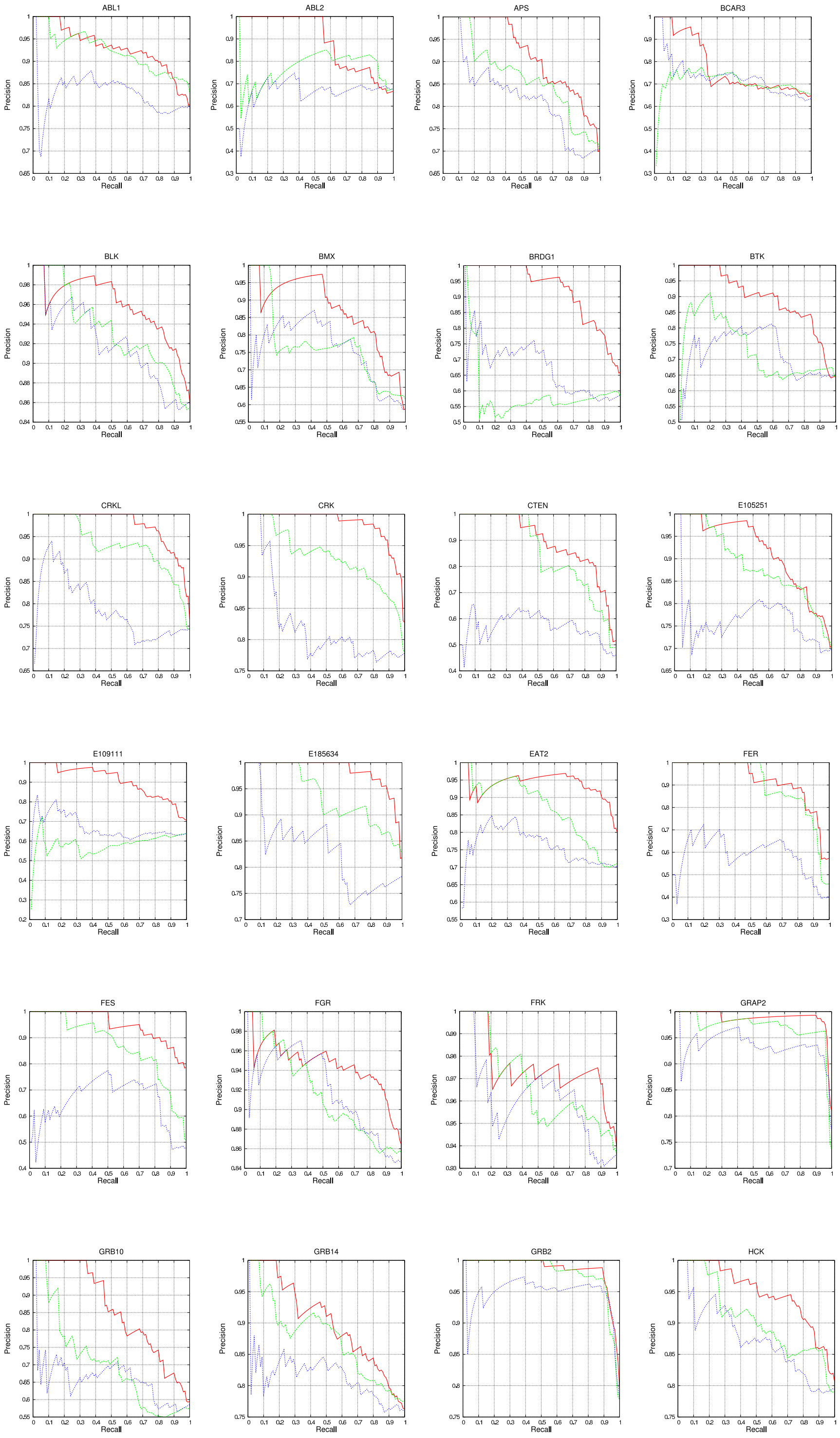

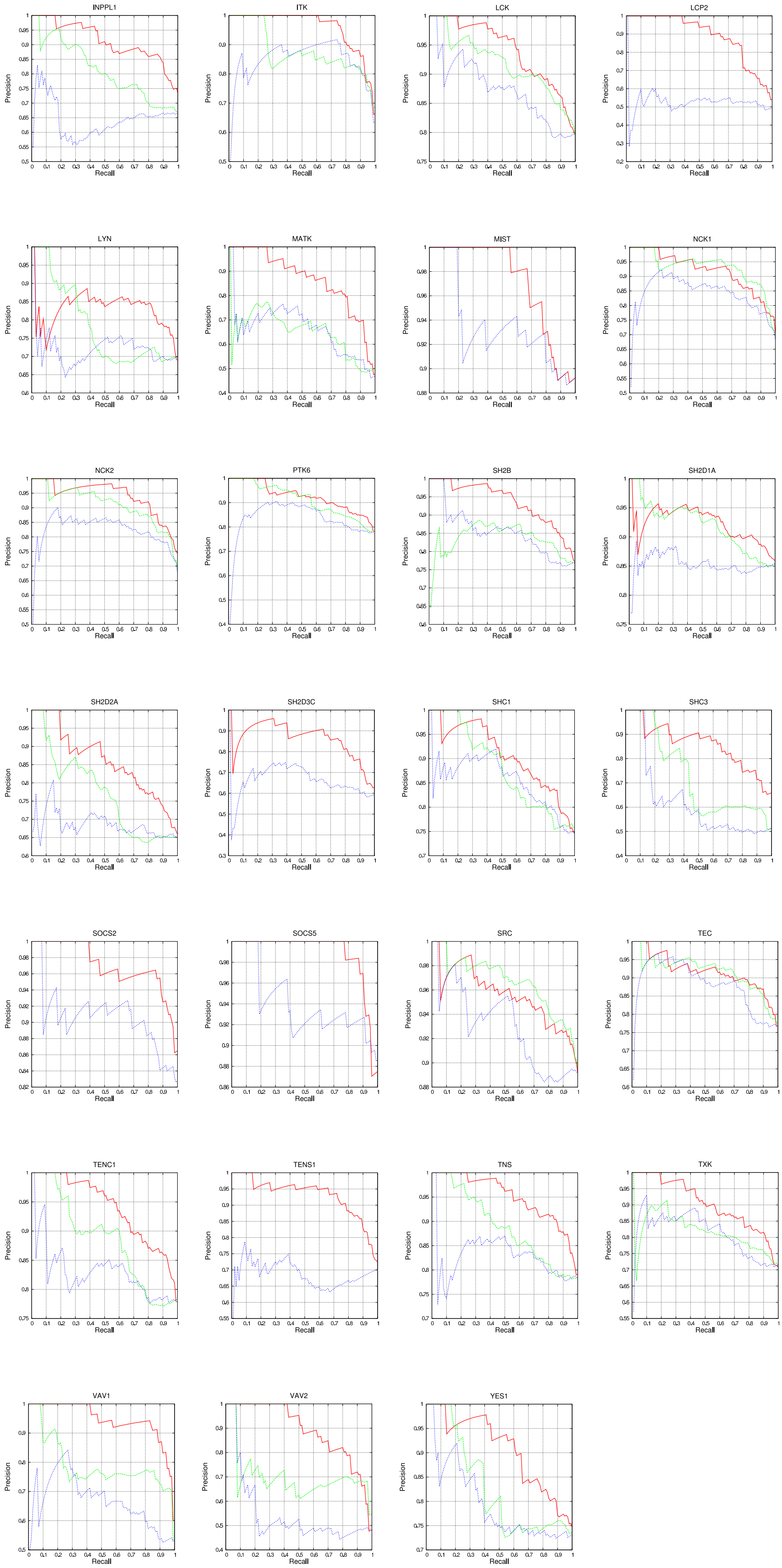
INPPL1
ITK
LCK
LCP2
LYN
MATK
MIST
NCK1
NCK2
PTK6
SH2B
SH2D1A
SH2D2A
SH2D3C
SHC1
SHC3
SOCS2
SOCS5
SRC
TEC
TENC1
TENS1
TNS
TXK
VAV1
VAV2
YES1
Precision
Recall
