# Supplementary material for: Semi-Supervised Prediction of SH2-Peptide Interactions from Imbalanced High-Throughput Data
Source: PLoS One. 2013 May 17;8(5):e62732. doi: 10.1371/journal.pone.0062732 (PMC3656881; doi:10.1371/journal.pone.0062732)
Supplement: Figure S4 — Binding and non-binding energy comparison. AUC ROC comparison of binding and non-binding energy for two different microarray data using energy based model. (PDF) [file pone.0062732.s004.pdf]

Figure S4: **Binding and non-binding energy comparison with different microarray data** AUC ROC of the dataset II and dataset III derived by the energy model [1]. Indicating the AUR ROC of the experiments and clearly showing the AUR ROC of dataset II, 0.97 (red line) is much higher that AUR ROC of dataset III, 0.56 (green dashed line). This result is probably due to some over-training issues.

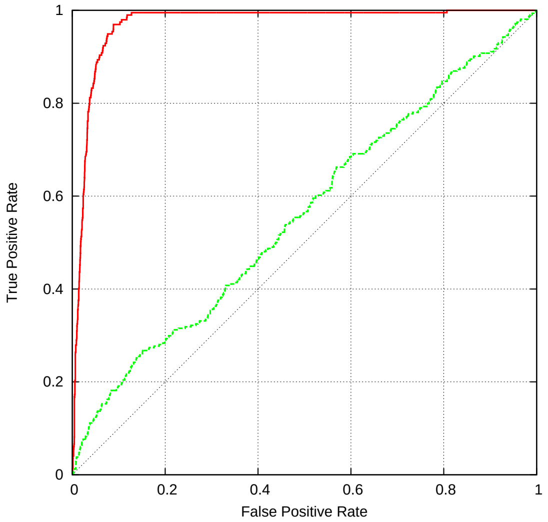

References

1. Wunderlich Z, Mirny LA (2009) Using genome-wide measurements for computational prediction of SH2-peptide interactions 37: 4629-41.
