# Supplementary material for: Semi-Supervised Prediction of SH2-Peptide Interactions from Imbalanced High-Throughput Data
Source: PLoS One. 2013 May 17;8(5):e62732. doi: 10.1371/journal.pone.0062732 (PMC3656881; doi:10.1371/journal.pone.0062732)
Supplement: Table S1 — Imbalanced dataset. Imbalanced level for confirmed presence or absence of peptide interactions with techniques 51 SH2 domains of this study. (PDF) [file pone.0062732.s005.pdf]

Table S1: **Imbalanced level for confirmed presence or absence of peptide interactions with techniques 51 SH2 domains of this study:** #int is the total number of interactions, #pos is the total number of positive interactions, #neg is the total number of negative interactions and ratio of the positive and negative interactions.

Domains	# int	# pos	# neg	ratio
ABL1	222	178	44	4:1
ABL2	61	40	21	2:1
APS	194	136	58	2:1
BCAR3	145	92	53	2:1
BLK	278	238	40	6:1
BMX	137	80	57	1:1
BRDG1	146	85	61	1:1
BTK	160	103	57	2:1
CRKL	177	131	46	3:1
CRK	204	158	46	3:1
CTEN	103	47	56	1:1
E105251	204	143	61	2:1
E109111	156	99	57	1:1
E185634	93	73	20	4:1
EAT2	200	141	59	2:1
FER	99	39	60	1:2
FES	115	55	60	1:1
FGR	328	278	50	6:1
FRK	284	266	18	15:1
GRAP2	223	164	59	3:1
GRB10	126	73	53	1:1
GRB14	243	185	58	3:1
GRB2	247	193	54	3:1
HCK	275	218	57	4:1
INPPL1	184	123	61	2:1
ITK	120	77	43	2:1
LCK	273	217	56	4:1
LCP2	120	59	61	1:1
LYN	154	102	52	2:1
MATK	113	53	60	1:1
MIST	93	83	10	8:1
NCK1	160	109	51	2:1
NCK2	149	101	48	2:1
PTK6	266	206	60	3:1
SH2B	237	182	55	4:1
SH2D1A	394	337	57	6:1
SH2D2A	172	112	60	2:1
SH2D3C	130	76	54	1:1
SHC1	202	151	51	3:1
SHC3	114	58	56	1:1

Domains	# int	# pos	# neg	ratio
SOCS2	116	96	20	5:1
SOCS5	80	70	10	7:1
SRC	373	333	40	8:1
TEC	214	165	49	3:1
TENC1	252	197	55	4:1
TENS1	177	124	53	2:1
TNS	261	205	56	4:1
TXK	188	133	55	2:1
VAV1	115	59	56	1:1
VAV2	89	40	49	1:1
YES1	149	109	40	3:1
