# Supplementary material for: Semi-Supervised Prediction of SH2-Peptide Interactions from Imbalanced High-Throughput Data
Source: PLoS One. 2013 May 17;8(5):e62732. doi: 10.1371/journal.pone.0062732 (PMC3656881; doi:10.1371/journal.pone.0062732)
Supplement: Table S2 — Comparison of linear and non-linear kernel. AUC ROC and AUC PR comparison of linear and non-linear kernel for each SH2 domain. (PDF) [file pone.0062732.s006.pdf]

Table S2: **Comparison of linear and non-linear kernel**
We compare the AUC PR and AUC ROC of linear and non-linear kernel for each SH2 domain. The better performers are in bold. The table indicates that the non-linear (i.e. polynomial in our case) kernel performs better than linear kernel.

**Domains**	**AUC PR**		**AUC ROC**	
	**Linear**	**Non-linear**	**Linear**	**Non-linear**
ABL1	0.916	**0.934**	0.757	**0.781**
ABL2	0.885	**0.914**	0.761	**0.798**
APS	0.909	**0.923**	0.801	**0.823**
BCAR3	0.785	**0.786**	**0.642**	0.636
BLK	0.965	**0.972**	0.833	**0.857**
BMX	0.885	**0.912**	0.834	**0.859**
BRDG1	0.925	**0.942**	0.886	**0.907**
BTK	0.872	**0.906**	0.773	**0.826**
CRK	0.982	**0.985**	0.943	**0.947**
CRKL	0.970	**0.976**	0.921	**0.931**
CTEN	0.841	**0.910**	0.865	**0.903**
E105251	0.923	**0.926**	0.824	**0.825**
E109111	0.903	**0.912**	**0.855**	0.846
E185634	**0.988**	0.985	**0.954**	0.940
EAT2	0.944	**0.953**	0.895	**0.918**
FER	0.874	**0.928**	0.914	**0.956**
FES	0.953	**0.966**	0.958	**0.970**
FGR	0.948	**0.959**	0.802	**0.820**
FRK	0.976	0.976	0.761	**0.767**
GRAP2	0.981	**0.987**	0.961	**0.972**
GRB10	0.845	**0.879**	0.783	**0.808**
GRB14	0.878	**0.905**	0.710	**0.739**
GRB2	0.979	**0.987**	0.937	**0.956**
HCK	0.939	**0.952**	0.810	**0.838**
INPPL1	0.902	**0.922**	0.835	**0.857**
ITK	0.955	**0.961**	0.903	**0.919**
LCK	**0.947**	0.943	**0.822**	0.804
LCP2	0.872	**0.892**	0.851	**0.879**
LYN	0.856	**0.890**	0.792	**0.825**
MATK	**0.870**	0.846	**0.868**	0.846
MIST	**0.974**	0.966	**0.788**	0.739
NCK1	0.903	**0.923**	0.818	**0.853**
NCK2	0.924	**0.949**	0.857	**0.894**
PTK6	0.909	**0.935**	0.771	**0.803**
SH2B	0.934	**0.939**	0.804	**0.824**
SH2D1A	0.931	**0.938**	0.725	**0.737**
SH2D2A	0.843	**0.878**	0.742	**0.777**
SH2D3C	0.865	**0.887**	0.825	**0.832**
SHC1	0.902	**0.915**	0.763	**0.781**
SHC3	0.866	**0.870**	**0.866**	0.855

**Domains**	**AUC PR**		**AUC ROC**	
	**Linear**	**Non-linear**	**Linear**	**Non-linear**
SOCS2	0.969	**0.980**	0.873	**0.915**
SOCS5	0.989	**0.991**	0.921	**0.936**
SRC	0.959	0.959	**0.756**	0.740
TEC	0.907	**0.922**	0.781	**0.791**
TENC1	0.930	**0.941**	0.794	**0.810**
TENS1	0.923	**0.935**	0.842	**0.852**
TNS	0.938	**0.954**	0.807	**0.848**
TXK	0.889	**0.905**	0.784	**0.793**
VAV1	0.946	**0.947**	**0.942**	0.930
VAV2	0.902	**0.903**	0.891	**0.904**
YES1	0.885	**0.924**	0.721	**0.800**
